# Bone morphogenetic protein 2 promotes human trophoblast cell invasion by upregulating N-cadherin via non-canonical SMAD2/3 signaling

**DOI:** 10.1038/s41419-017-0230-1

**Published:** 2018-02-07

**Authors:** Hong-Jin Zhao, Christian Klausen, Yan Li, Hua Zhu, Yan-Ling Wang, Peter C. K. Leung

**Affiliations:** 1 Shandong Provincial Hospital affiliated to Shandong University, Ji’nan, PR China 250021; 2 Department of Obstetrics and Gynaecology, BC Children’s Hospital Research Institute, University of British Columbia, Vancouver, British Columbia Canada V5Z 4H4; 3 State Key Laboratory of Stem Cell and Reproductive Biology, Institute of Zoology, Chinese Academy of Sciences, Beijing, China 100101

## Abstract

BMP2 expression is spatiotemporally correlated with embryo implantation and is crucial for endometrial decidualization and fertility in mice. BMP2 has been reported to increase the mesenchymal adhesion molecule N-cadherin and enhance cell invasion in cancer cells; moreover, studies suggest that N-cadherin promotes placental trophoblast invasion. However, whether BMP2 can promote trophoblast cell invasion during placentation remains unknown. The objective of our study was to investigate the effects of BMP2 on human trophoblast cell invasion and the involvement of N-cadherin and SMAD signaling. Primary and immortalized (HTR8/SVneo) cultures of human extravillous trophoblast (EVT) cells were used as study models. Treatment with recombinant human BMP2 increased HTR8/SVneo cell transwell Matrigel invasion as well as N-cadherin mRNA and protein levels, but had no significant effect on cell proliferation. Likewise, BMP2 treatment enhanced primary human EVT cell invasion and N-cadherin production. Basal and BMP2-induced invasion were attenuated by small interfering RNA-mediated downregulation of N-cadherin in both HTR8/SVneo and primary EVT cells. Intriguingly, BMP2 induced the phosphorylation/activation of both canonical SMAD1/5/8 and non-canonical SMAD2/3 signaling in HTR8/SVneo and primary EVT cells. Knockdown of SMAD2/3 or common SMAD4 totally abolished the effects of BMP2 on N-cadherin upregulation in HTR8/SVneo cells. Upregulation of SMAD2/3 phosphorylation and N-cadherin were totally abolished by type I receptor activin receptor-like kinases 2/3 (ALK2/3) inhibitor DMH1; moreover, knockdown of ALK2 or ALK3 inhibited N-cadherin upregulation. Interestingly, activation of SMAD2/3 and upregulation of N-cadherin were partially attenuated by ALK4/5/7 inhibitor SB431542 or knockdown of ALK4, but not ALK5. Our results show that BMP2 promotes trophoblast cell invasion by upregulating N-cadherin via non-canonical ALK2/3/4-SMAD2/3-SMAD4 signaling.

## Introduction

Extravillous cytotrophoblasts (EVTs) derived from villous cell columns invade into the maternal uterine wall for proper placentation and successful establishment of human pregnancy^1^. Insufficient trophoblast invasion is thought to contribute to several pregnancy complications, such as preeclampsia that affects 2–8% of pregnancies worldwide and is a leading cause of maternal mortality^2,3^. Therefore, it is essential to better understand the regulation of trophoblast invasion and identify key signaling molecules underlying this process in order to improve the diagnosis and treatment of these conditions.

Transforming growth factor-β (TGF-β) superfamily members exert a variety of regulatory effects on trophoblast invasion during embryo implantation. TGF-β1 suppresses EVT invasiveness by downregulating matrix metalloproteinase 9 and vascular endothelial cadherin^4,5^, whereas activin A promotes invasion by upregulating N-cadherin and matrix metalloproteinase 2^6,7^. However, there have been no reports about the effects of bone morphogenetic proteins (BMPs) on trophoblast cell invasion. BMPs are the biggest subfamily of the TGF-β superfamily and consist of over 20 isoforms. Their roles in organogenesis are conserved from insects to humans, and they may also play key roles in placentation^8,9^. Classically, BMPs function by activating heterotetrameric complexes of type I ALK (activin receptor-like kinases) and type II transmembrane serine–threonine kinase receptors, which subsequently phosphorylate and activate receptor-regulated SMAD1/5/8. Phosphorylated SMAD1/5/8 then binds to common SMAD4 and translocate into the nucleus to mediate BMP-regulated gene expression^10–12^.

In situ hybridization studies in mice have demonstrated that, unlike Bmp4, 5, 6, 7, 8a, and 8b, uterine expression of Bmp2 was spatiotemporally correlated with embryo implantation, suggesting important functions for Bmp2 during implantation and early placentation^13^. Conditional knockout and in vitro studies revealed that Bmp2 was crucial for endometrial decidualization and fertility in mice and humans^14,15^. Although the decidua produces BMP2, it is not known whether BMP2 regulates trophoblast cell invasiveness. However, pro-invasive effects of BMP2 have been reported in breast, colon, gastric, and pancreatic cancer cell lines, and likely involve aspects of EMT including upregulation of N-cadherin^16–21^.

Cadherins are transmembrane proteins mediating calcium-dependent cell–cell adhesion with the cytoplasmic domain interacting with catenin and elements of the actin cytoskeleton^22^. N-cadherin is a mesenchymal adhesion molecule and its upregulation has been shown to correlate with invasive properties of cancer cells^23^. Studies suggest that trophoblast invasion shares several features with tumor cell invasion, although the latter lacks strict physiological control. Interestingly, switching expression from E-cadherin (epithelial marker) to N-cadherin (mesenchymal marker) is involved in trophoblast differentiation along the invasive pathway and failure to switch is associated with insufficient invasion and abnormal placentation^24,25^. However, it is not known whether BMP2 can promote human trophoblast cell invasion or whether such an effect involves the upregulation of N-cadherin.

In the present study, we have examined the effects of BMP2 on human trophoblast cell invasion and the regulation and involvement of N-cadherin in these effects. Our results show that BMP2 treatment enhances trophoblast cell invasion and N-cadherin expression. Furthermore, the pro-invasive effects of BMP2 on trophoblast invasion are mediated by upregulating N-cadherin via non-canonical SMAD2/3-SMAD4-dependent signaling.

## Results

### BMP2 enhances human trophoblast cell invasion

**Fig. 1:**
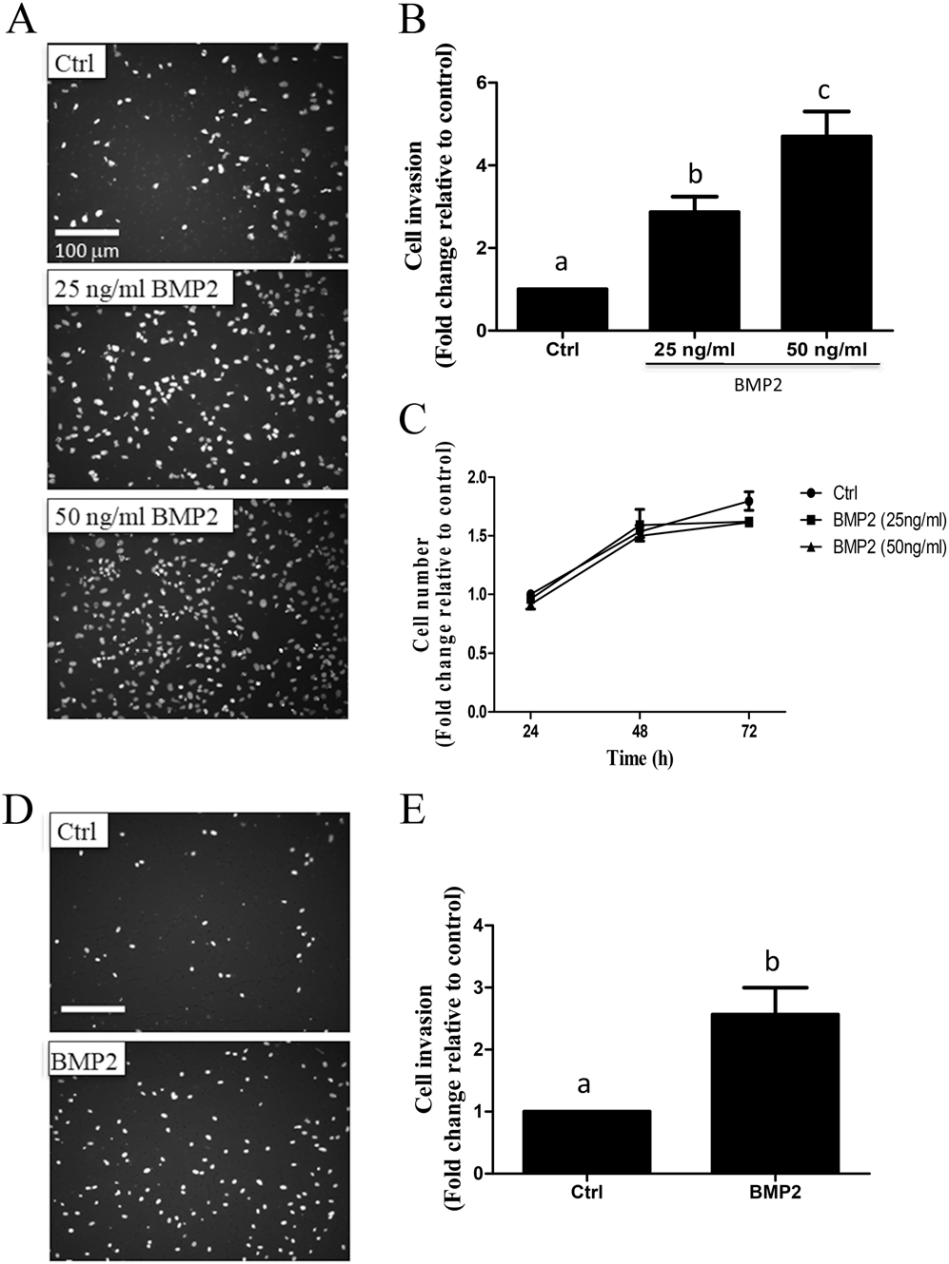
BMP2 increases HTR8/SVneo and primary human EVT cell invasion. **a**, **b** HTR8/SVneo cells were treated with vehicle (Ctrl) or BMP2 (25 or 50 ng/mL) and cell invasiveness was examined by Matrigel-coated transwell assay. Representative images of the invasion assay ((**a**); scale bar 100 μm) and summarized quantitative results (**b**) are shown separately (*n* = 3). **c** HTR8/SVneo cells were seeded in 24-well plates and treated every 24 h with vehicle or BMP2 (25 or 50 ng/mL) for a total of 72 h. Cell viability was determined by MTT assay at 24, 48, and 72 h after BMP2 treatment (*n* = 3). **d**, **e** Primary human EVT cells were treated with or without 25 ng/mL BMP2 and cell invasiveness was examined by Matrigel-coated transwell assay. Representative images of the invasion assay ((**d**); scale bar 100 μm) and combined quantitative results (**e**) are shown separately (*n* = 5). Results are displayed as the mean ± SEM of at least three independent experiments and values without common letters are significantly different (*P* < 0.05)

To determine the effects of BMP2 on trophoblast cell invasion, Matrigel-coated transwell invasion assays were carried out following treatment of HTR8/SVneo cells with 25 or 50 ng/mL recombinant human BMP2. The results showed that BMP2 significantly increased HTR8/SVneo cell invasion in a concentration-dependent manner (Figs. 1a,b). To examine whether the enhanced trophoblast invasion induced by BMP2 might be influenced by any effect of BMP2 on cell viability, MTT assays were performed and the results confirmed that treatment with 25 or 50 ng/mL BMP2 for as long as 72 h had no significant effect on HTR8/SVneo cell viability (Fig. 1c). Similar to HTR8/SVneo cells, treatment with 25 ng/mL BMP2 significantly enhanced cell invasion in primary human EVT cells (Figs. 1d,e). Since 25 ng/mL BMP2 could promote human trophoblast cell invasion significantly, this concentration of BMP2 was used to treat HTR8/SVneo cell line as well as primary trophoblast cells in the following experiments.

### BMP2 upregulates trophoblast N-cadherin levels

**Fig. 2:**
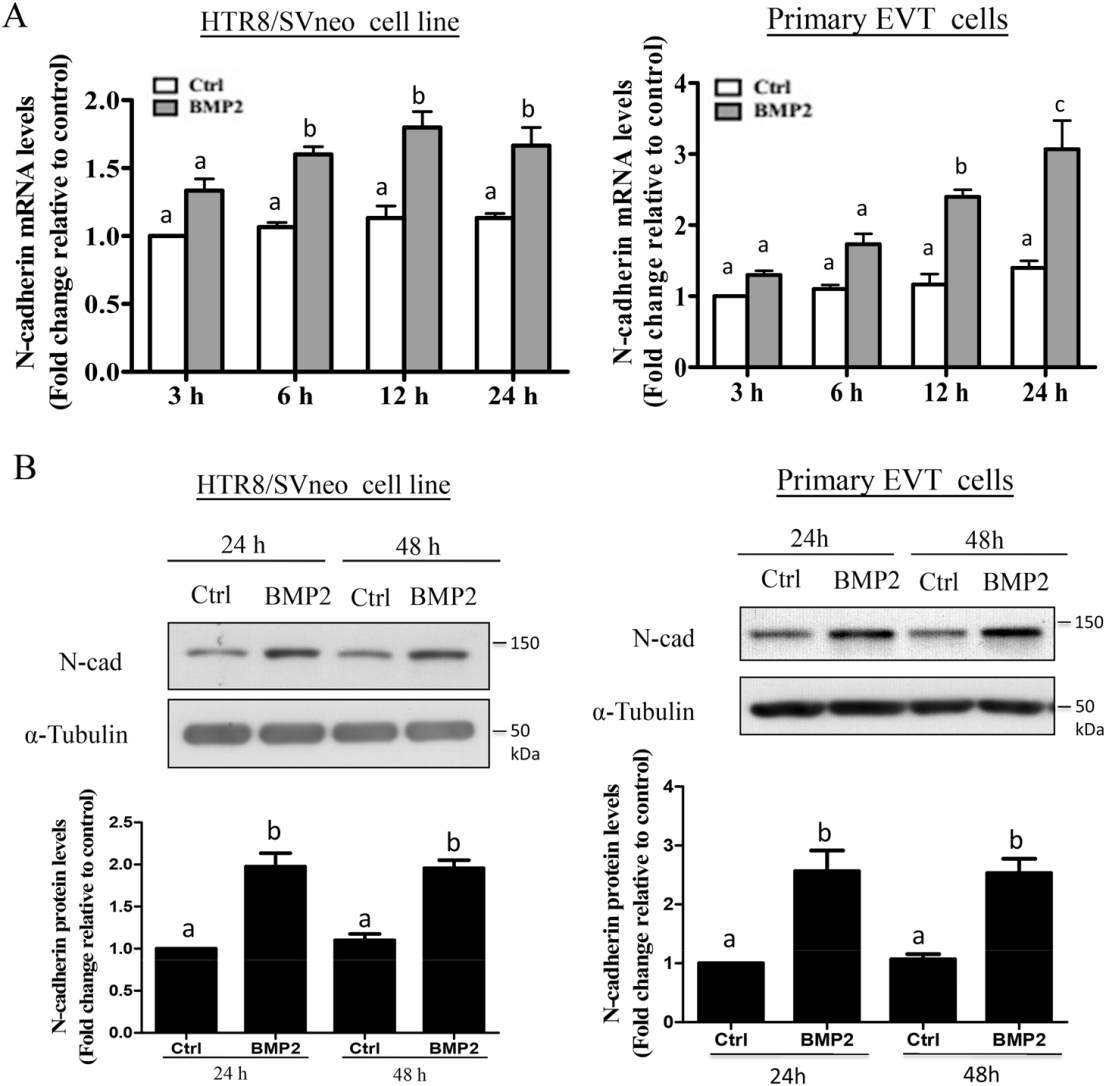
BMP2 increases N-cadherin mRNA and protein levels in HTR8/SVneo and primary human EVT cells. **a** HTR8/SVneo cells (left panel) or primary EVT cells (right panel) were treated with vehicle (Ctrl) or 25 ng/mL BMP2 for different lengths of time (3, 6, 12, or 24 h), and N-cadherin mRNA levels were examined by RT-qPCR with GAPDH as the reference gene. **b** HTR8/SVneo cells (left panel) or primary EVT cells (right panel) were treated with or without 25 ng/mL BMP2 every 24 h for 48 h, and N-cadherin protein levels were analyzed by western blot and normalized to α-tubulin. Results are displayed as the mean ± SEM of at least three independent experiments and values without common letters are significantly different (*P* < 0.05)

To examine the effects of BMP2 on N-cadherin expression, HTR8/SVneo and primary EVT cells were treated with 25 ng/mL BMP2 for 3, 6, 12, 24, or 48 h. RT-qPCR results showed that BMP2 treatment increased N-cadherin mRNA levels in both HTR8/SVneo and primary EVT cells between 6 and 24 h (Fig. 2a). Similarly, western blot analysis revealed upregulation of N-cadherin protein levels following treatment of HTR8/SVneo and primary EVT cells with BMP2 for 24 and 48 h (Fig. 2b).

### N-cadherin upregulation contributes to BMP2-induced trophoblast cell invasion

**Fig. 3:**
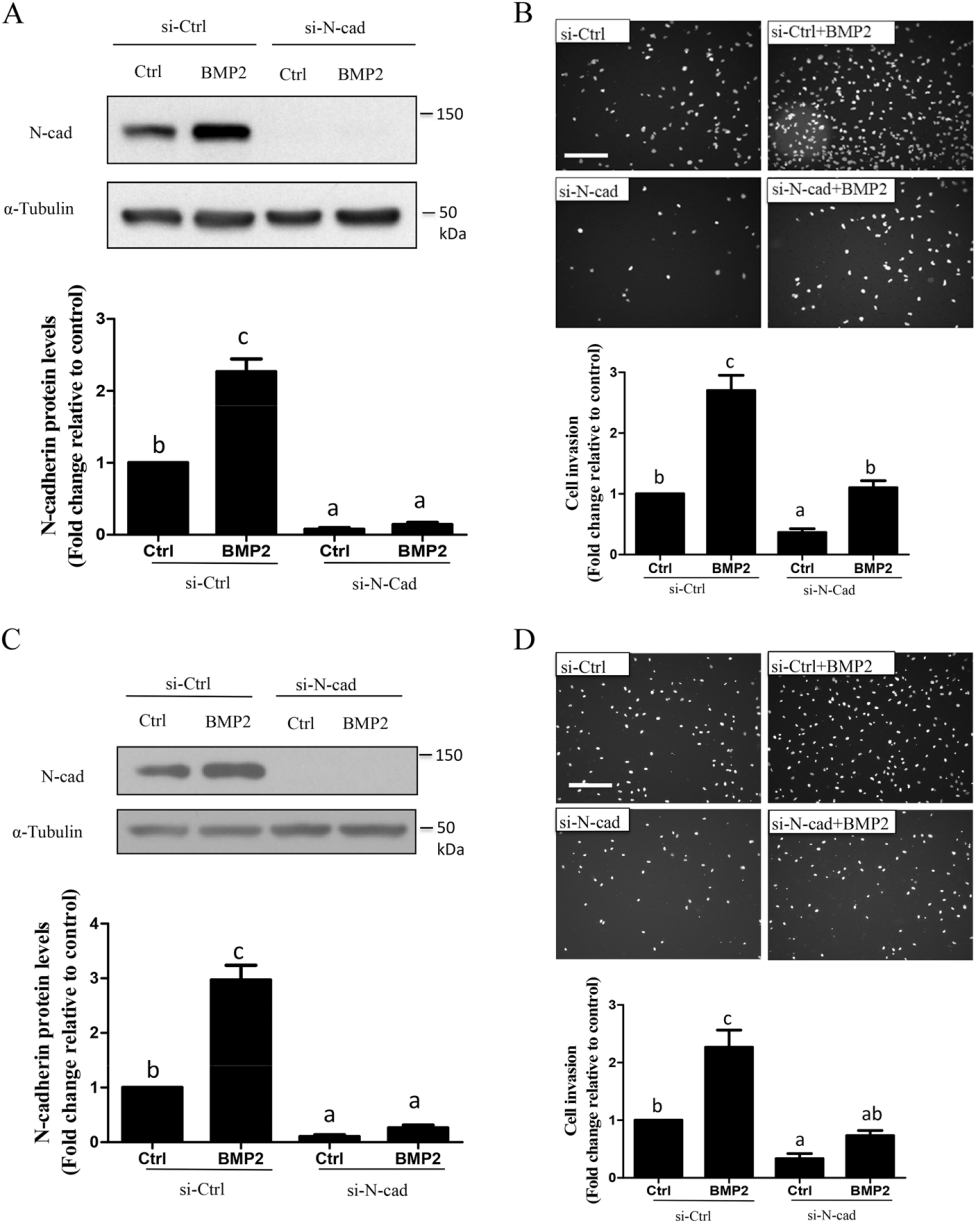
Knockdown of N-cadherin attenuates basal and BMP2-induced trophoblast cell invasion. **a–d** HTR8/SVneo or primary human EVT cells were transfected for 48 h with 25 nM non-targeting control siRNA (si-Ctrl) or 25 nM siRNA targeting N-cadherin (si-N-cad) prior to treatment with vehicle (Ctrl) or 25 ng/mL BMP2. Western blot was used to measure N-cadherin protein levels in HTR8/SVneo (**a**) and primary EVT (**c**) cells 24 h after treatment with BMP2. **b**, **d** Matrigel-coated transwell assays were used to examine the effects of N-cadherin knockdown on BMP2-induced invasion in HTR8/SVneo (**b**; *n* = 3) and primary EVT (**d**) cells (*n* = 5). Summarized quantitative results are expressed as the mean ± SEM of at least three independent experiments and values without common letters are significantly different (*P* < 0.05)

To determine whether N-cadherin upregulation is involved in BMP2-induced trophoblast cell invasion, we performed siRNA-mediated knockdown of N-cadherin. Pretreatment of HTR8/SVneo cells with siRNA targeting N-cadherin for 48 h suppressed both basal and BMP2-induced N-cadherin protein levels (Fig. 3a). Matrigel-coated transwell invasion assays revealed that depletion of N-cadherin attenuated both basal and BMP2-induced HTR8/SVneo cell invasion (Fig. 3b). Importantly, the contribution of N-cadherin to basal and BMP2-induced cell invasion was also confirmed in first-trimester primary human EVT cells (Figs. 3c,d).

### Non-canonical SMAD2/3 signaling is required for BMP2-induced N-cadherin upregulation

**Fig. 4:**
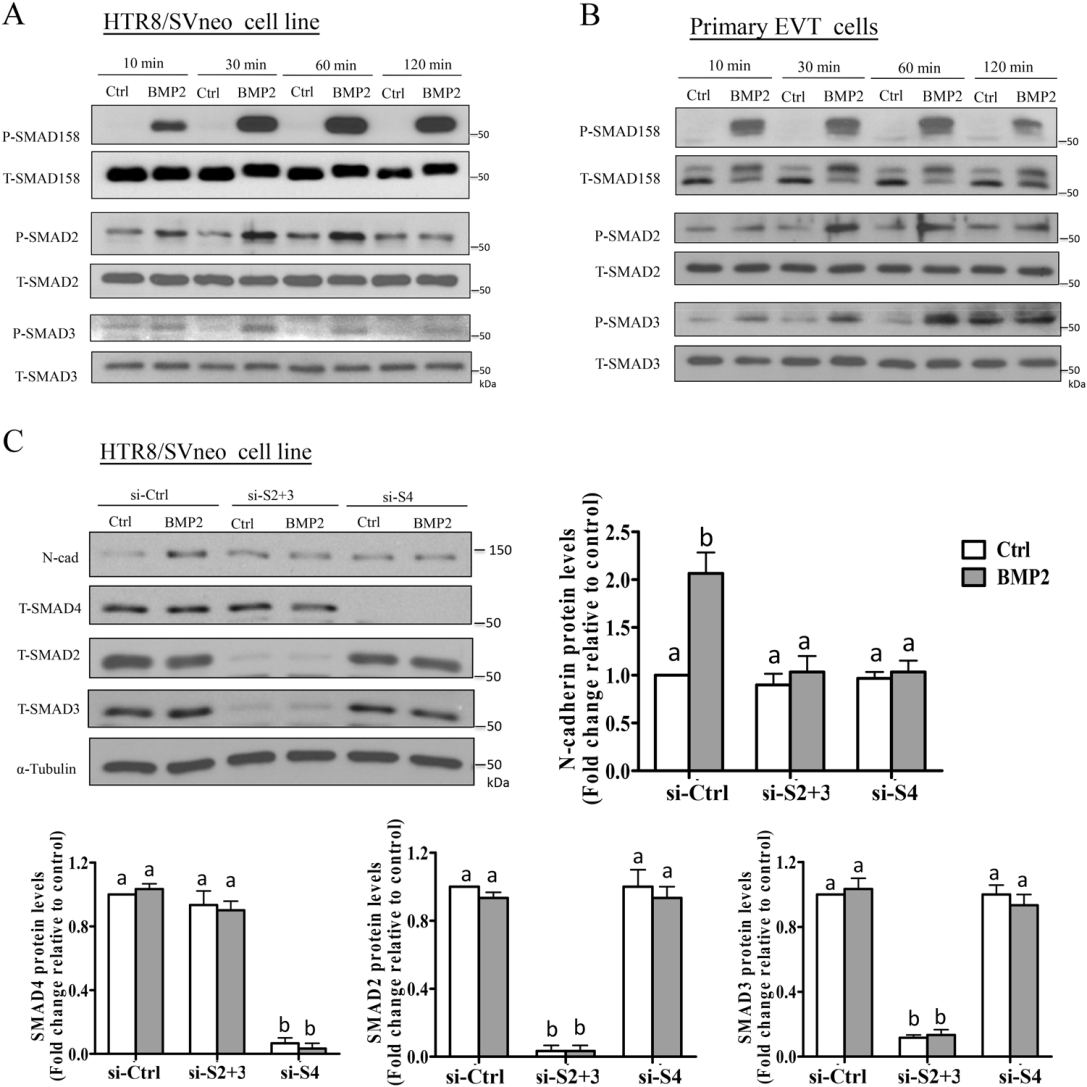
Non-canonical SMAD2/3 signaling mediates the upregulation of N-cadherin by BMP2. **a**, **b** HTR8/SVneo (**a**) and primary EVT (**b**) cells were treated with vehicle (Ctrl) or 25 ng/mL BMP2 for different lengths of time (10, 30, 60, or 120 min). Levels of phosphorylated SMAD1/5/8 (P-SMAD1/5/8), SMAD2 (P-SMAD2), and SMAD3 (P-SMAD3) were examined by western blot with corresponding phospho-specific antibodies. Membranes were stripped and reprobed with antibodies for total SMAD1/5/8 (T-SMAD1/5/8), SMAD2 (T-SMAD2), and SMAD3 (T-SMAD3). **c** HTR8/SVneo cells were transfected for 48 h with 25 nM non-targeting control siRNA (si-Ctrl), 25 nM siRNA targeting SMAD2 + SMAD3 (si-S2 + 3), or 25 nM siRNA targeting SMAD4 (si-S4) prior to treatment with vehicle (Ctrl) or 25 ng/mL BMP2 for 24 h. Protein levels of N-cadherin, T-SMAD2, T-SMAD3, and T-SMAD4 were examined by western blot (*n* = 4). Summarized quantitative results are displayed as the mean ± SEM of at least three independent experiments and values without common letters are significantly different (*P* < 0.05)

BMPs exert their biological effects primarily via canonical SMAD1/5/8-SMAD4 signaling; however, several reports have demonstrated the involvement of non-canonical SMAD2/3-SMAD4 and/or SMAD-independent signaling^12,18,26–28^. Thus, we used western blot to measure changes in the phosphorylation/activation of SMAD1/5/8 or SMAD2/3 following treatment of HTR8/SVneo or primary EVT cells with BMP2. BMP2 treatment increased not only the phosphorylation of canonical SMAD1/5/8 as expected, but also induced the phosphorylation of non-canonical SMAD2/3 in HTR8/SVneo cells (Fig. 4a). Confirmatory experiments performed with primary EVT cells likewise showed that BMP2 treatment increased the phosphorylation of both SMAD1/5/8 and SMAD2/3 (Fig. 4b). To investigate the involvement of SMAD signaling in BMP2-induced N-cadherin production, we first performed knockdown of common SMAD4. As shown in Fig. 3c, the stimulatory effect of BMP2 on N-cadherin protein level was abolished by knockdown of common SMAD4 in HTR8/SVneo cells. Next, we performed combined knockdown of SMAD2/3 since we have previously shown that SMAD2/3 mediates N-cadherin upregulation in response to activin A in human trophoblast cells^6^. Interestingly, depletion of SMAD2/3 completely abolished the upregulation of N-cadherin by BMP2 in HTR8/SVneo cells (Fig. 3c), suggesting that non-canonical SMAD2/3 signaling is essential for BMP2-induced N-cadherin production.

### BMP2 increases N-cadherin production via both BMP (ALK2/3) and activin (ALK4) type I receptors

**Fig. 5:**
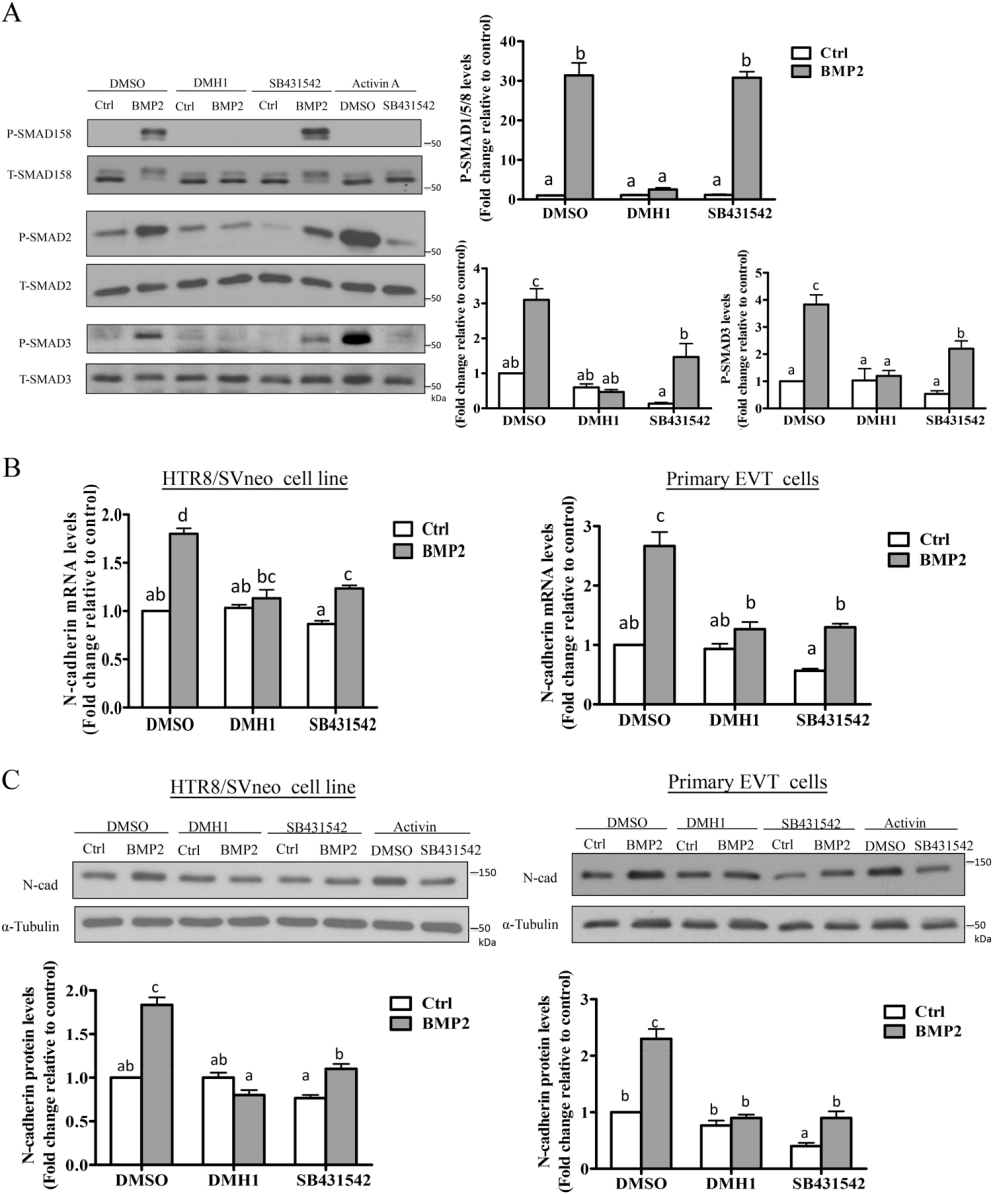
BMP and activin/TGF-β type I receptors contribute to BMP2-induced phosphorylation of SMAD2/3 and upregulation of N-cadherin. **a** HTR8/SVneo cells were pretreated for 1 h with vehicle control (DMSO), 1 μM DMH1 (ALK2/3 inhibitor), or 10 μM SB431542 (ALK4/5/7 inhibitor) prior to treatment for 30 min with vehicle (Ctrl), 25 ng/mL BMP2, or 50 ng/mL activin A. Protein levels of phosphorylated SMAD1/5/8 (P-SMAD1/5/8), SMAD2 (P-SMAD2), and SMAD3 (P-SMAD3) were examined by western blot with corresponding phospho-specific antibodies. Membranes were stripped and reprobed with antibodies for total SMAD1/5/8 (T-SMAD1/5/8), SMAD2 (T-SMAD2), and SMAD3 (T-SMAD3). **b** HTR8/SVneo and primary EVT cells were pretreated for 1 h with DMH1 (1 μM) or SB431542 (10 μM) prior to treatment for 12 h with or without 25 ng/mL BMP2, and N-cadherin mRNA levels were measured by RT-qPCR. **c** HTR8/SVneo and primary EVT cells were pretreated for 1 h with DMH1 (1 μM) or SB431542 (10 μM) prior to treatment for 24 h with BMP2 (25 ng/mL) or activin A (50 ng/mL), and N-cadherin protein levels were analyzed by western blot. Results are expressed as the mean ± SEM of at least three independent experiments and values without common letters are significantly different (*P* < 0.05)

BMPs generally activate SMAD1/5/8 signaling via BMP type I receptors ALK2, ALK3, and/or ALK6, whereas activins/TGF-βs induce the activation of SMAD2/3 via ALK4, ALK5, or ALK7. To determine which ALKs are involved in the activation of non-canonical SMAD2/3 and upregulation of N-cadherin by BMP2, we first used a pharmacological approach with selective inhibitors of BMP or activin/TGF-β type I receptors. Western blot was used to measure BMP2-induced SMAD phosphorylation in HTR8/SVneo cells following pretreatment with the ALK2/3 inhibitor DMH1^29^ or the ALK4/5/7 inhibitor SB431542^30^. HTR8/SVneo cells were also treated with activin A as a positive control since it has previously been shown to upregulate N-cadherin and induce SMAD2/3 signaling, both of which can be totally blocked by pretreatment with SB431542^6^. As expected, BMP2-induced phosphorylation of SMAD1/5/8 was abolished by pretreatment with DMH1 but was not influenced by SB431542 pretreatment (Fig. 5a). Interestingly, DMH1 also abolished BMP2-induced phosphorylation of SMAD2 and SMAD3, whereas pretreatment with SB431542 was partially inhibitory (Fig. 5a). In contrast, activin A-induced SMAD2/3 phosphorylation was totally blocked by pretreatment with SB431542 (Fig. 5a) as previously reported^6^. In both HTR8/SVneo and primary EVT cells, the upregulation of N-cadherin mRNA (Fig. 5b) and protein (Fig. 5c) levels by BMP2 was completely inhibited by treatment with DMH1 and partially inhibited by treatment with SB431542.

**Fig. 6:**
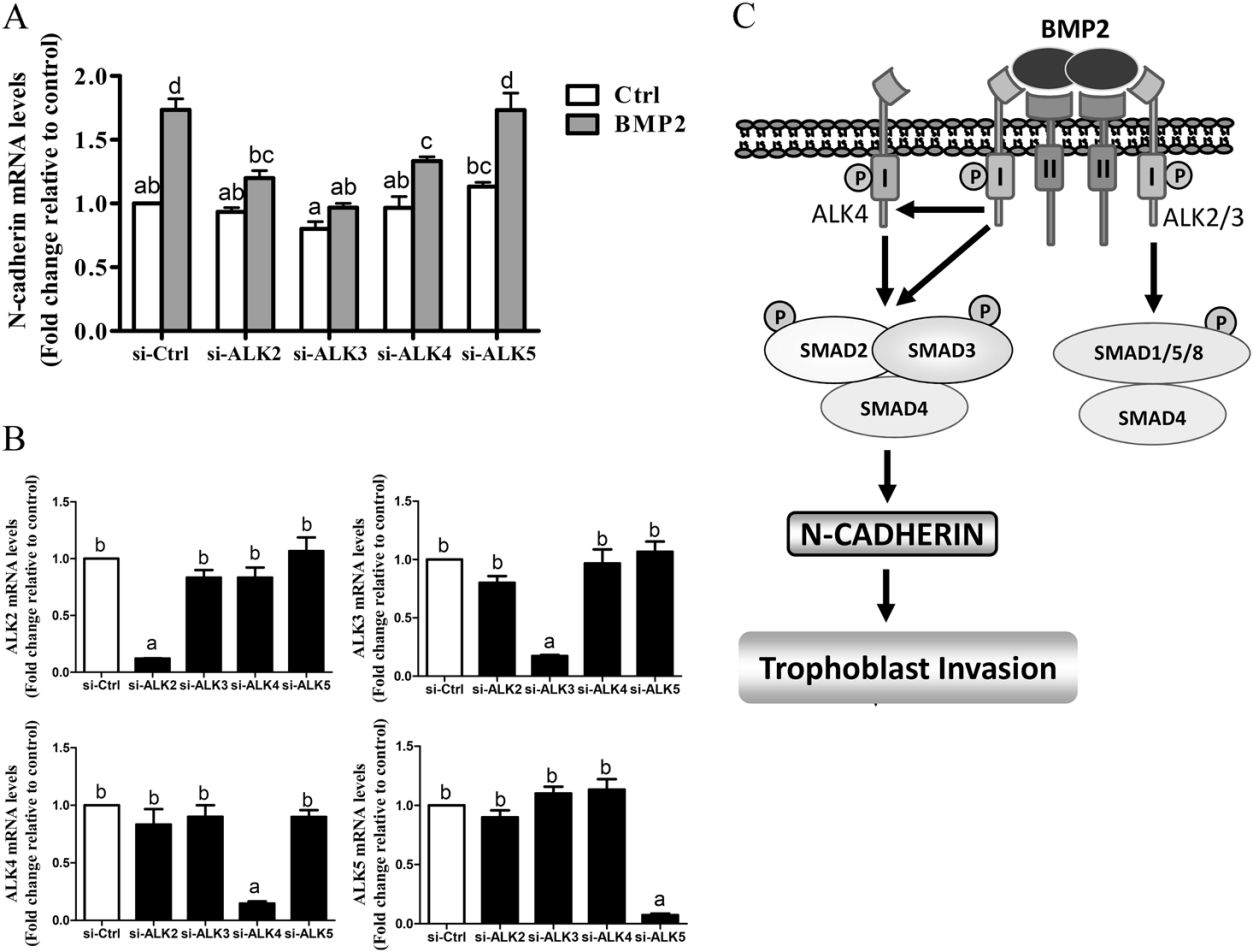
BMP (ALK2/3) and activin (ALK4) type I receptors mediate the upregulation of N-cadherin by BMP2. **a**, **b** HTR8/SVneo cells were transfected for 48 h with 25 nM non-targeting control siRNA (si-Ctrl) or 25 nM siRNA targeting ALK2 (si-ALK2), ALK3 (si-ALK3), ALK4 (si-ALK4) or ALK5 (si-ALK5). Cells were treated for a further 12 h with vehicle (Ctrl) or 25 ng/mL BMP2 and RT-qPCR were used to measure the mRNA levels of N-cadherin (**a**) and ALKs (**b**). Summarized quantitative results are displayed as the mean ± SEM of three independent experiments and values without common letters are significantly different (*P* < 0.05). **c** Proposed model of the signaling pathway mediating BMP2-induced N-cadherin upregulation and increased human trophoblast cell invasion. BMP2 binds to a complex of type I and II receptors leading to the activation of both canonical SMAD1/5/8 and non-canonical SMAD2/3 signaling. Activation of receptor complexes containing ALK2, ALK3, and ALK4 leads to the phosphorylation of SMAD2/3, which complexes with common SMAD4 and translocates into the nucleus to increase the transcription of N-cadherin, which promotes human trophoblast cell invasion. BMP2 activates canonical SMAD1/5/8 signaling via ALK2/3; however, the functional consequences of this signaling require further investigation

Next, we used siRNA-mediated knockdown approach to determine which ALKs are involved in the upregulation of N-cadherin by BMP2. HTR8/SVneo cells were pretreated for 48 h with siRNA targeting ALK2, ALK3, ALK4, or ALK5 prior to treatment with BMP2 for 12 h. BMP2-induced increases in N-cadherin mRNA levels were abolished by knockdown of ALK3 and partially inhibited by depletion of ALK2 or ALK4 (Fig. 6a). In contrast, downregulation of ALK5 did not affect the upregulation of N-cadherin by BMP2 (Fig. 6a). Together, these results suggest that BMP2 increases N-cadherin production via ALK2, ALK3, and ALK4 in human trophoblast cells.

## Discussion

We propose that, besides its role in endometrial decidualization^14,15^, BMP2 may contribute to embryo implantation and early placental development by promoting trophoblast invasion. In particular, we have shown that BMP2 promotes primary and immortalized human EVT cell invasion by upregulating N-cadherin. Moreover, we demonstrate that, whereas BMP2 activates both canonical SMAD1/5/8 and non-canonical SMAD2/3 signaling, only SMAD2/3 signaling seems to be required for its upregulation of N-cadherin. In vivo studies of the putative functions of BMP2 in endometrial decidulization have been performed in mice using a conditional knockout approach because total knockout of Bmp2 or Bmp receptors in mice leads to embryonic lethality or severe defects in cardiac development^31,32^. Progesterone receptor locus-guided conditional knockout of BMP type II receptor (*Bmpr2 cKO*) in the uterus revealed impairments in both endometrial decidulization and trophoblast invasion^32^. At the time it was suggested that the defects in trophoblast invasion were simply due to impaired decidulization. However, it should be noted that mouse trophoblast cells also express progesterone receptor^33^ and that some of this impairment could reflect direct effects in trophoblast cells due to *Bmpr2* haploinsufficiency in a majority of the embryos produced by *Bmpr2 cKO* mice. BMPR2 binds BMPs as well as some growth differentiation factors so it remains to be determined exactly what the in vivo functions of BMP2 are in early placental development. However, we have detected high levels of BMP2 mRNA in primary human EVT cells (data not shown), which suggests that the effects of BMP2 on trophoblast invasion could be mediated in an autocrine as well as paracrine manner.

During differentiation to invasive EVTs, trophoblasts of epithelial lineage must undergo epithelial–mesenchymal transition (EMT) to acquire the motility and invasive potential necessary for remodeling of the decidua and spiral arteries^24,25^. Defects in this process are associated with preeclampsia, a common pregnancy complication characterized, in part, by shallow trophoblast invasion^3^. EMT is a fundamental process that is important for embryonic development as well as cancer progression; however, its involvement in invasive trophoblast differentiation is yet to be fully delineated. BMP2 has been implicated in EMT-like processes in both physiological and pathological contexts. For example, BMP2 was shown to be critical for cardiac cushion formation by inducing endocardial EMT^34^. In cancer cells, BMP2 increases cell invasion via PI3K/AKT signaling and induces EMT-like changes in gene expression, such as downregulation of E-cadherin and upregulation of SNAIL or SLUG^20,21^. In addition to increasing N-cadherin and cell invasion, treatment of trophoblastic HTR8/SVneo cells with BMP2 increased the expression of several EMT-associated genes, including matrix metalloproteinase 2, SNAIL, and SLUG (Supplementary Figure 1). Induction of these EMT-associated genes could act in parallel with N-cadherin to increase HTR8/SVneo cell invasion, and might account for inability of N-cadherin knockdown to completely inhibit BMP2-induced HTR8/SVneo cell invasion. However, BMP2 has also been reported to suppress colorectal cancer cell growth by inhibiting proliferation and inducing apoptosis^35^. Besides, BMP2 has been shown to inhibit liver cancer cell migration and growth by downregulating PI3K/AKT signaling^36^. Thus, BMP2 can exert varying, and even opposing, effects on cancer cells depending on type and context. In EVT cells the effects of BMP2 appear to be largely restricted to the promotion of invasiveness since we did not observe any significant changes in cell viability. Nevertheless, future studies are required to investigate the effects of BMP2 on other trophoblast cell functions, especially in cytotrophoblast or syncytiotrophoblast cell populations.

BMP2-induced activation or inactivation of SMAD-independent signaling, especially PI3K/AKT and MAPK pathways, has been implicated in the promotion or suppression of cancer progression^36,37^. However, our study revealed that short-term treatment with BMP2 (<2 h) had no effects on AKT or ERK1/2 phosphorylation/activation (data not shown). Rather, BMP2 treatment induced the activation of both canonical SMAD1/5/8 and non-canonical SMAD2/3 signaling, although only the latter mediated N-cadherin upregulation in human trophoblast cells. In a study of 46 normal and cancer cell lines, BMP2 was shown to induce SMAD2/3 signaling preferentially in embryonic and transformed cells^18^. Our findings of BMP2-induced SMAD2/3 phosphorylation in human EVT cells are in agreement since trophoblast cells are derived from the blastocyst trophectoderm. Interestingly, Holtzhausen et al. also noted greater co-expression of genes responsive to SMAD1/5/8 and SMAD2/3 in breast and liver cancer samples compared to normal tissues, suggesting cooperation between these two SMAD signaling pathways in cancer^18^. Our findings do not exclude a role for SMAD1/5/8 signaling in BMP2-induced trophoblast invasion. Indeed, BMP2-induced upregulation of furin, which is a pro-protein convertase that can directly activate membrane type 1 MMP (MT1-MMP)^38^, is blocked by SMAD4 knockdown but not by combined knockdown of SMAD2/3, suggesting regulation by SMAD1/5/8-SMAD4 signaling (Supplementary Figure 2). Trophoblast cells are often thought of as pseudomalignant because they share a number of features with cancer cells. Future studies investigating functional cooperation between canonical SMAD1/5/8 and non-canonical SMAD2/3 signaling in human trophoblast cells will be of great interest.

TGF-β superfamily members signal in a SMAD-dependent manner to regulate trophoblast invasion during embryo implantation. However, in contrast to the classical notion of BMP signaling, we have shown that BMP2 enhances EVT cell invasion by upregulating N-cadherin via non-canonical SMAD2/3 signaling. Generally, BMPs are thought to bind ALK2/3/6 type I receptors and activate SMAD1/5/8, whereas TGF-βs/activins usually bind ALK4/5/7 to induce SMAD2/3. However, recent studies in cancer, pituitary, and ovarian cells have demonstrated BMP-induced activation of SMAD2/3 signaling via several mechanisms. Holtzhausen et al. used a variety of techniques to show that BMP2 could induce heterodimeric type I receptor complexes composed of ALK5/7 and ALK3/6, which were capable of phosphorylating SMAD2/3 in a variety of cancer cell lines^18^. Direct activation of SMAD2 and SMAD3 by ALK3 has been demonstrated in BMP2-treated melanoma and pituitary gonadotrope cells, respectively^27,28^. In human ovarian granulosa cells, BMP4 was shown to activate SMAD2 via ALK3 and ALK4/5, whereas it activated SMAD3 via ALK3 and ALK4^26^. Together, our inhibitor and knockdown results suggest that BMP2-induced increases in SMAD2/3 phosphorylation and N-cadherin production are mediated by both ALK2 and ALK3. In contrast to previous studies in cancer and pituitary gonadotrope cells^18,28,39^, our study in trophoblast cells is the first to implicate ALK2 in BMP2-induced non-canonical SMAD2/3 signaling. Our inhibitor and knockdown results also show that ALK4, but not ALK5, is partially involved in the SMAD2/3-dependent upregulation of N-cadherin by BMP2. These results are unique compared to previous studies in cancer cells implicating only ALK5 or both ALK4/5 in BMP2-induced SMAD2/3 signaling^18,28^. In agreement with the classical notion of BMP signaling, our SB431542 results show that only ALK2/3 are involved in the activation of canonical SMAD1/5/8 signaling. Future studies are required to investigate in greater detail the precise roles of SMAD2/3 *vs*. SMAD1/5/8 signaling in the effects of BMP2 on human trophoblast invasion as well as other biological responses.

Overall, our study demonstrates for the first time that BMP2 promotes human trophoblast invasion in addition to its well-established roles in endometrial decidulization. The pro-invasive effects of BMP2 are mediated, in part, by upregulation of N-cadherin via non-canonical SMAD2/3-SMAD4 signaling. BMP2 induces SMAD2/3 signaling via ALK2, ALK3, and ALK4, whereas it activates canonical SMAD1/5/8 signaling via ALK2/3 (Fig. 6c). These findings deepen our understanding of the roles of BMP2 in placentation and provide insight into the molecular mechanisms of human trophoblast invasion.

## Materials and Methods

### Culture of HTR8/SVneo human EVT cell line

The HTR8/SVneo simian virus 40 large T-antigen-immortalized first-trimester human EVT cell line was kindly provided by Dr. P.K. Lala (Western University, Canada)^40^. Cells were cultured in DMEM (Life Technologies) supplemented with 10% (vol/vol) fetal bovine serum (FBS), 100 U/mL penicillin, and 100 μg/mL streptomycin (Life Technologies). Cultures were maintained at 37 °C in a humidified atmosphere with 5% CO_2_ in air.

### Primary human EVT isolation and culture

This study was approved by the Research Ethics Board of the University of British Columbia and all women provided informed written consent. Seventeen first-trimester human placentas (6–10 weeks' gestation) were collected from women undergoing elective termination of pregnancy. Primary human EVT cells were isolated from chorionic villous explants as previously described and cultured at 37 °C in a humidified 5% CO_2_/air atmosphere^6,41^. Briefly, the placenta villi tips were finely minced and cultured for 3–4 days in flasks with DMEM (Life Technology) supplemented with 10% (vol/vol) FBS, 100 U/mL penicillin, and 100 μg/mL streptomycin. Non-attached pieces were removed and attached villous tissue fragments were cultured for another 10–14 days to allow for EVT outgrowth. EVT cells were subsequently separated from villous explants by trypsinization. The purity of EVT cell cultures was verified by immunocytochemical staining for cytokeratin-7 and human leukocyte antigen G (HLA-G). Only cultures showing more than 99% positive staining for cytokeratin-7 and HLA-G were used in this study. Each experiment performed with primary EVT cells was replicated with cells from five different placentas.

### Reagents and antibodies

Recombinant human BMP2 and DMH1 were obtained from R&D Systems. SB431542 (catalog no. S4317) was purchased from Sigma-Aldrich. Mouse monoclonal anti-cytokeratin-7 (OV-TL 12/30) and anti-HLA-G (4H84) were purchased from Millipore and Exbio, respectively. Mouse monoclonal anti-N-cadherin antibody (catalog no. 610920) was obtained from BD Biosciences. Rabbit monoclonal anti-phospho-SMAD2 (Ser^465/467^; 138D4), mouse monoclonal anti-SMAD2 (L16D3), rabbit monoclonal anti-phospho-SMAD3 (Ser^423/425^; C25A9), rabbit monoclonal SMAD3 (C67H9), rabbit polyclonal anti-SMAD4 (catalog no. 9515), and anti-phospho-SMAD1 (Ser^463/465^)/SMAD5 (Ser^463/465^)/SMAD8 (Ser^426/428^; catalog no. 9511) were purchased from Cell Signaling Technology. Rabbit polyclonal anti-SMAD1/5/8 (N-18; catalog no. sc-6031-R) and mouse monoclonal anti-α-Tubulin (B-5-1-2; catalog no. sc-23948) were obtained from Santa Cruz Biotechnology. Horseradish peroxidase-conjugated goat anti-mouse IgG and goat anti-rabbit IgG were obtained from Bio-Rad Laboratories.

### Matrigel-coated transwell invasion assay

Trophoblast cell invasiveness was examined using Corning Biocoat Growth Factor Reduced Matrigel Invasion Chamber (pore size, 8 μm; catalog no. 354483) as per the guidelines for use. Briefly, cells were pretreated with vehicle or BMP2 (25 or 50 ng/mL) for 20 min. Then, each insert was seeded with 5 × 10^4^ cells suspended in 250 μL vehicle/BMP2-containing DMEM medium supplemented with 0.1% (vol/vol) FBS and 750 μL DMEM medium with 10% (vol/vol) FBS was added to the lower chamber. After 24 h incubation, cells in each insert were retreated with vehicle or BMP2 (25 or 50 ng/mL) and incubated for a further 12 h (36 h in total). At the end of the experiment, non-invading cells were removed from the upper side of the membrane and cells on the lower side were fixed with cold methanol (−20 °C) and air-dried. Cell nuclei were stained with Hoechst 33258 (Sigma-Aldrich) and imaged with a fluorescent microscope followed by analysis with Image-J software. Duplicate inserts were used for each individual experiment, and five random microscopic fields were counted per insert.

### Reverse transcription quantitative real-time PCR

Total RNA was extracted with TRIzol Reagent (Life Technologies) as per the manufacturer’s instructions. Reverse transcription was carried out with 2 μg RNA, random primers, and Moloney murine leukemia virus reverse transcriptase (Promega) in a final volume of 20 μL. SYBR Green or TaqMan reverse transcription quantitative real-time PCR (RT-qPCR) was performed on an Applied Biosystems 7300 Real-Time PCR System equipped with 96-well optical reaction plates. Each 20 μL SYBR Green RT-qPCR reaction contained 1 × SYBR Green PCR Master Mix (Applied Biosystems), 20 ng cDNA, and 250 nM of each specific primer. The primers used were: N-cadherin (*CDH2*), 5′-GGACAGTTCCTGAGGGATCA-3′ (forward) and 5′-GGATTGCCTTCCATGTCTGT-3′ (reverse); glyceraldehyde-3-phosphate dehydrogenase (*GAPDH*), 5′-GAGTCAACGGATTTGGTCGT-3′ (forward) and 5′- GACAAGCTTCCCGTTCTCAG-3′ (reverse). The specificity of each assay was validated by dissociation curve analysis and agarose gel electrophoresis of PCR products. TaqMan gene expression assays for *BMP2* (catalog no. Hs00154192_m1), *ALK2* (catalog no. Hs00153836_m1), *ALK3* (catalog no. Hs01034913_g1), *ALK4* (catalog no. Hs00244715_m1), *ALK5* (catalog no. Hs00610320_m1), and *GAPDH* (catalog no. Hs02758991_g1) were purchased from Applied Biosystems. Each 20 μL TaqMan RT-qPCR reaction contained 1 × TaqMan Gene Expression Master Mix (Applied Biosystems), 20 ng cDNA, and 1 × specific TaqMan assay containing primers and probe. Each sample was assayed in triplicate and a mean value from at least three independent experiments was used for relative quantification of mRNA levels by the comparative Cq method with GAPDH as the reference gene and using the formula 2^−ΔΔCq^.

### Western blot analysis

Cells were lysed in ice-cold lysis buffer (Cell Signaling Technology) with added protease inhibitor cocktail (Sigma-Aldrich). Extracts were centrifuged at 13,000 r.p.m. for 15 min at 4 °C and supernatant protein concentrations were determined using the DC Protein Assay (Bio-Rad Laboratories) with Bovine Serum Albumin (BSA) as the standard. Equal amounts of protein were separated by standard Tris-glycine SDS-PAGE and electrotransferred to Polyvinylidene fluoride (PVDF) membranes. Membranes were blocked with Tris-buffered saline containing 5% (wt/vol) non-fat dry milk for 1 h and then immunoblotted overnight at 4°C with specific primary antibodies diluted in Tris-buffered saline with 5% (wt/vol) non-fat dried milk and 0.1% (vol/vol) Tween-20. After incubation with appropriate horseradish peroxidase-conjugated secondary antibody for 1 h at room temperature, signals were detected with enhanced chemiluminescent or SuperSignal West Femto chemiluminescent substrates (Thermo Fisher) and CL-XPosure film (Thermo Fisher). Membranes were stripped with stripping buffer (62.5 mM Tris-HCl (pH 6.8), 100 mM β-mercaptoethanol, and 2% (wt/vol) SDS) at 50 °C for 30 min and reprobed as described above with antibodies against α-tubulin, SMAD2, SMAD3, or SMAD1/5/8. Densitometric quantification was performed using Image-Pro Plus software with α-tubulin, SMAD2, SMAD3 or SMAD1/5/8 for normalization.

### Small interfering RNA transfection

Cells at ~50% confluency were transfected for 48 h with 25 nM ON-TARGET*plus* NON-TARGETING*pool* small interfering RNA (siRNA) or ON-TARGET*plus* SMART*pool* siRNA targeting human N-cadherin (L-011605-00-0005), SMAD2 (L-003561-00-0005), SMAD3 (L-020067-00-0005), SMAD4 (L-003902-00-0005), ALK2 (L-004924-00-0005), ALK3 (L-004933-00-0005), ALK4 (L-004925-00-0005), or ALK5 (L-003929-00-0005; Dharmacon) using Lipofectamine RNAiMAX (Life Technologies) according to the manufacturer’s instructions. Knockdown efficiency was assessed by RT-qPCR or western blot analysis.

### MTT assay

MTT (3-(4,5-dimethylthiazol-2-yl)-2,5-diphenyltetrazolium bromide; Sigma) assay was used to assess cell viability. HTR8/SVneo cells were plated in 24-well plates (1.2 × 10^4^ cells/well in 1 mL 10% FBS medium) and incubated for 12 h before starvation with 0.1% FBS medium for another 24 h. Cells were then treated every 24 h with or without BMP2 in medium containing 0.1% FBS for total 72 h. Cells were incubated with 0.5 mg/mL MTT for 4 h after which the medium was replaced with 1 mL dimethylsulfoxide and absorbances were measured at 490 nm using a microplate reader.

### Statistical analysis

Results are presented as the mean ± SEM of at least three independent experiments. Multiple group comparisons were analyzed by one-way ANOVA followed by Newman–Keuls test using PRISM software (GraphPad Software Inc). Means were considered significantly different if *P* < 0.05 and are indicated by different letters.

## Electronic supplementary material

Supplemental figure legends

Supplementary Figure 1

Supplementary Figure 2

## References

[1] Knofler M (2010). Critical growth factors and signalling pathways controlling human trophoblast invasion. Int. J. Dev. Biol..

[2] Chaddha V, Viero S, Huppertz B, Kingdom J (2004). Developmental biology of the placenta and the origins of placental insufficiency. Semin. Fetal Neonatal Med..

[3] Lim KH (1997). Human cytotrophoblast differentiation/invasion is abnormal in preeclampsia. Am. J. Pathol..

[4] Lash GE (2005). Inhibition of trophoblast cell invasion by TGFB1, 2, and 3 is associated with a decrease in active proteases. Biol. Reprod..

[5] Cheng JC, Chang HM, Leung PC (2013). Transforming growth factor-beta1 inhibits trophoblast cell invasion by inducing Snail-mediated down-regulation of vascular endothelial-cadherin protein. J. Biol. Chem..

[6] Li Y, Klausen C, Cheng JC, Zhu H, Leung PC (2014). Activin A, B, and AB increase human trophoblast cell invasion by up-regulating N-cadherin. J. Clin. Endocrinol. Metab..

[7] Li Y, Klausen C, Zhu H, Leung PC (2015). Activin A increases human trophoblast invasion by inducing SNAIL-mediated MMP2 up-regulation through ALK4. J. Clin. Endocrinol. Metab..

[8] Massague J, Chen YG (2000). Controlling TGF-beta signaling. Genes Dev..

[9] Hogan BL (1996). Bone morphogenetic proteins: multifunctional regulators of vertebrate development. Genes Dev..

[10] Zhang Y, Feng X, We R, Derynck R (1996). Receptor-associated Mad homologues synergize as effectors of the TGF-beta response. Nature.

[11] Bragdon B (2011). Bone morphogenetic proteins: a critical review. Cell. Signal..

[12] Chang HM, Qiao J, Leung PC (2016). Oocyte-somatic cell interactions in the human ovary-novel role of bone morphogenetic proteins and growth differentiation factors. Hum. Reprod. Update.

[13] Paria BC (2001). Cellular and molecular responses of the uterus to embryo implantation can be elicited by locally applied growth factors. Proc. Natl Acad. Sci. USA.

[14] Lee KY (2007). Bmp2 is critical for the murine uterine decidual response. Mol. Cell Biol..

[15] Li Q (2007). Bone morphogenetic protein 2 functions via a conserved signaling pathway involving Wnt4 to regulate uterine decidualization in the mouse and the human. J. Biol. Chem..

[16] Kim BR (2015). BMP-2 induces motility and invasiveness by promoting colon cancer stemness through STAT3 activation. Tumour Biol..

[17] Yang Y, Yang C, Zhang J (2015). C23 protein meditates bone morphogenetic protein-2-mediated EMT via up-regulation of Erk1/2 and Akt in gastric cancer. Med. Oncol..

[18] Holtzhausen A (2014). Novel bone morphogenetic protein signaling through Smad2 and Smad3 to regulate cancer progression and development. FASEB J..

[19] Clement JH (2005). Bone morphogenetic protein 2 (BMP-2) induces in vitro invasion and in vivo hormone independent growth of breast carcinoma cells. Int. J. Oncol..

[20] Kang MH, Kim JS, Seo JE, Oh SC, Yoo YA (2010). BMP2 accelerates the motility and invasiveness of gastric cancer cells via activation of the phosphatidylinositol 3-kinase (PI3K)/Akt pathway. Exp. Cell Res..

[21] Chen X, Liao J, Lu Y, Duan X, Sun W (2011). Activation of the PI3K/Akt pathway mediates bone morphogenetic protein 2-induced invasion of pancreatic cancer cells Panc-1. Pathol. Oncol. Res..

[22] Berx G, van Roy F (2009). Involvement of members of the cadherin superfamily in cancer. Cold Spring Harb. Perspect. Biol..

[23] Cavallaro U (2004). N-cadherin as an invasion promoter: a novel target for antitumor therapy?. Curr. Opin. Investig. Drugs.

[24] Kokkinos MI, Murthi P, Wafai R, Thompson EW, Newgreen DF (2010). Cadherins in the human placenta--epithelial-mesenchymal transition (EMT) and placental development. Placenta.

[25] E Davies J (2016). Epithelial-mesenchymal transition during extravillous trophoblast differentiation. Cell Adh Migr..

[26] Zhang H (2016). Differential activation of non-canonical SMAD2/SMAD3 signaling by bone morphogenetic proteins causes disproportionate induction of hyaluronan production in immortalized human granulosa cells. Mol. Cell Endocrinol..

[27] Wang Y (2014). Bone morphogenetic protein 2 stimulates non-canonical SMAD2/3 signaling via the BMP type 1A receptor in gonadotrope-like cells: implications for FSH synthesis. Endocrinology.

[28] Murakami M, Kawachi H, Ogawa K, Nishino Y, Funaba M (2009). Receptor expression modulates the specificity of transforming growth factor-beta signaling pathways. Genes Cells.

[29] Hao J (2010). In vivo structure-activity relationship study of dorsomorphin analogues identifies selective VEGF and BMP inhibitors. ACS Chem. Biol..

[30] Inman GJ (2002). SB-431542 is a potent and specific inhibitor of transforming growth factor-beta superfamily type I activin receptor-like kinase (ALK) receptors ALK4, ALK5, and ALK7. Mol. Pharmacol..

[31] Zhang H, Bradley A (1996). Mice deficient for BMP2 are nonviable and have defects in amnion/chorion and cardiac development. Development.

[32] Nagashima T (2013). BMPR2 is required for postimplantation uterine function and pregnancy maintenance. J. Clin. Invest..

[33] Dai B (2003). Dual roles of progesterone in embryo implantation in mouse. Endocrine.

[34] Ma L, Lu MF, Schwartz RJ, Martin JF (2005). Bmp2 is essential for cardiac cushion epithelial-mesenchymal transition and myocardial patterning. Development.

[35] Zhang Y (2014). Bone morphogenetic protein 2 inhibits the proliferation and growth of human colorectal cancer cells. Oncol. Rep..

[36] Zheng Y (2014). Bone morphogenetic protein 2 inhibits hepatocellular carcinoma growth and migration through downregulation of the PI3K/AKT pathway. Tumour Biol..

[37] Zhang L (2016). BMP signaling and its paradoxical effects in tumorigenesis and dissemination. Oncotarget.

[38] Otto S (2016). A novel role of endothelium in activation of latent pro-membrane type 1 MMP and pro-MMP-2 in rat aorta. Cardiovasc. Res..

[39] Ho CC, Bernard DJ (2009). Bone morphogenetic protein 2 signals via BMPR1A to regulate murine follicle-stimulating hormone beta subunit transcription. Biol. Reprod..

[40] Graham CH (1993). Establishment and characterization of first trimester human trophoblast cells with extended lifespan. Exp. Cell Res..

[41] Irving JA (1995). Characteristics of trophoblast cells migrating from first trimester chorionic villus explants and propagated in culture. Placenta.

