# Supplementary material for: Bone morphogenetic protein 2 promotes human trophoblast cell invasion by upregulating N-cadherin via non-canonical SMAD2/3 signaling
Source: Cell Death Dis. 2018 Feb 7;9(2):174. doi: 10.1038/s41419-017-0230-1 (PMC5833391; doi:10.1038/s41419-017-0230-1)
Supplement: Supplementary file 1 — Supplemental figure legends [file 41419_2017_230_MOESM1_ESM.docx]

**Supplemental figure legends**

**Supplementary Figure 1.** BMP2 increases the expression of several EMT-associated genes in immortalized human trophoblast cells. HTR8/SVneo cells were treated with vehicle (Ctrl) or with 25 ng/mL BMP2 for different lengths of time (3, 6, 12 or 24 hours), and MMP2, SNAIL and SLUG mRNA levels were examined by RT-qPCR with GAPDH as the reference gene.

**Supplementary Figure 2.** Canonical SMAD1/5/8 signaling mediates the up-regulation of furin by BMP2. HTR8/SVneo cells were transfected for 48 h with 25 nM non-targeting control siRNA (si-Ctrl), 25 nM siRNA targeting SMAD2+SMAD3 (si-S2+3) or 25 nM siRNA targeting SMAD4 (si-S4) prior to treatment with vehicle (Ctrl) or 25 ng/mL BMP2 for 24 hours. Protein levels of furin, N-cadherin, T-SMAD2, T-SMAD3 and T-SMAD4 were examined by Western blot.
