# Supplementary figures and images for: Bone morphogenetic protein 2 promotes human trophoblast cell invasion by upregulating N-cadherin via non-canonical SMAD2/3 signaling
Source: Cell Death Dis. 2018 Feb 7;9(2):174. doi: 10.1038/s41419-017-0230-1 (PMC5833391; doi:10.1038/s41419-017-0230-1)

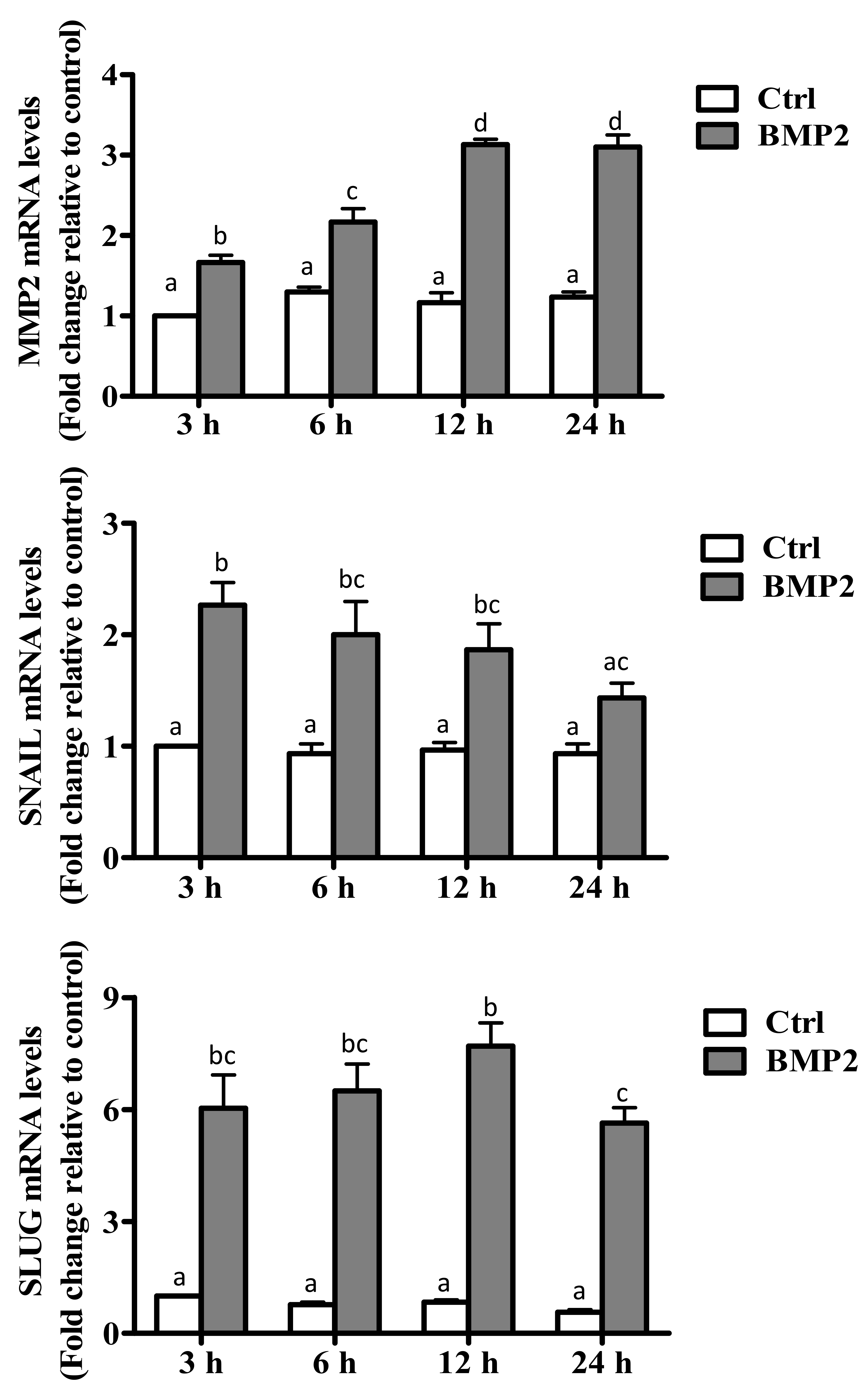

Supplement: Supplementary file 2 — Supplementary Figure 1 [file 41419_2017_230_MOESM2_ESM.tif]

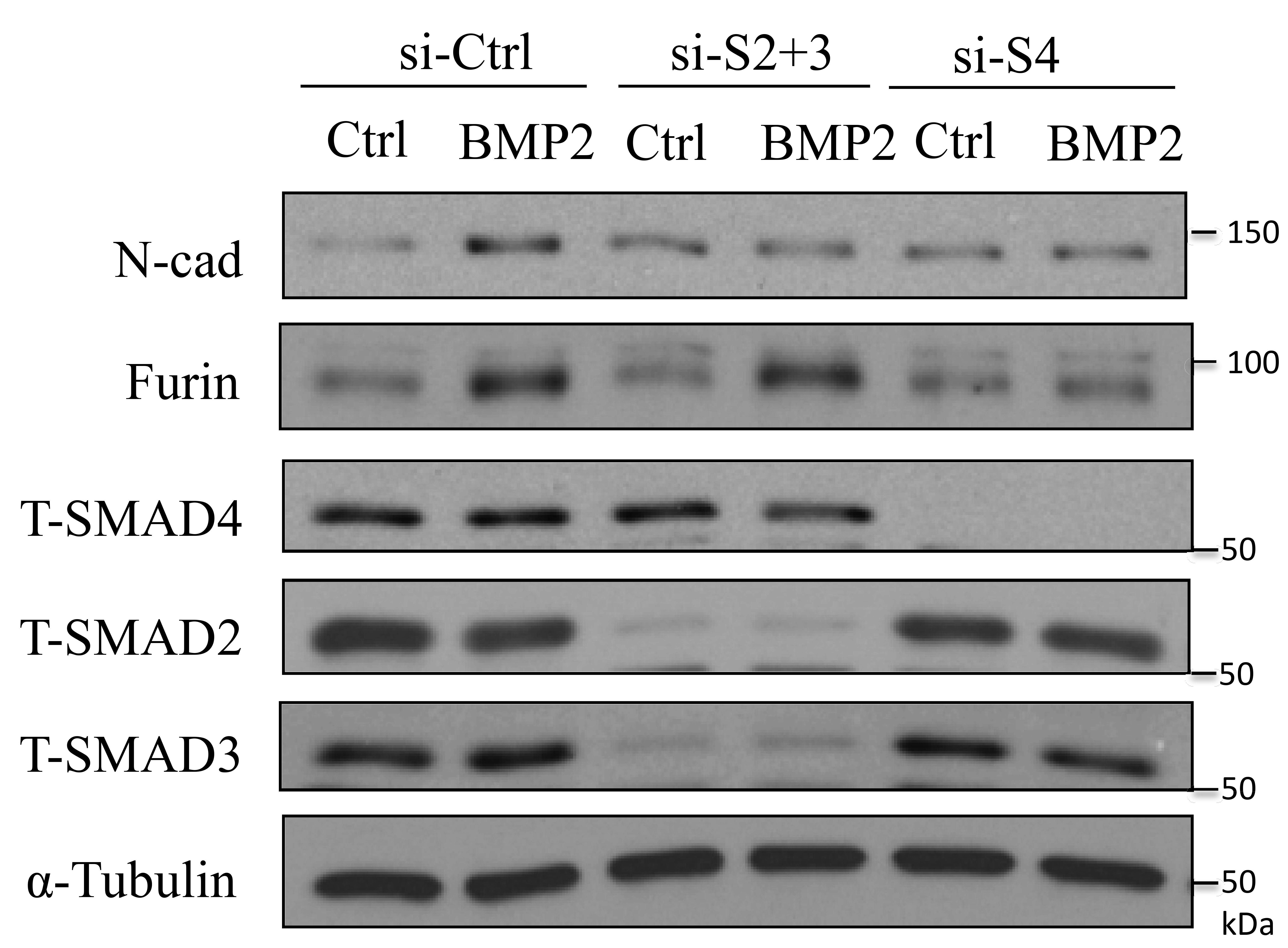

Supplement: Supplementary file 3 — Supplementary Figure 2 [file 41419_2017_230_MOESM3_ESM.tif]
